# Development and Validation of an HPLC-PDA Method for Biologically Active Quinonemethide Triterpenoids Isolated from *Maytenus chiapensis*

**DOI:** 10.3390/medicines6010036

**Published:** 2019-03-07

**Authors:** Vito Alessandro Taddeo, Ulises Guardado Castillo, Morena Lizette Martínez, Jenny Menjivar, Ignacio Antonio Jiménez, Marvin José Núñez, Isabel López Bazzocchi

**Affiliations:** 1 Instituto Universitario de Bio-Orgánica Antonio González, Departamento de Química Orgánica, Universidad de La Laguna, Avenida Astrofísico Francisco Sánchez 2, 38206 La Laguna, Tenerife, Spain; taddeovitoalessandro@gmail.com (V.A.T.); ignadiaz@ull.edu.es (I.A.J.); 2 Dipartimento di Farmacia, Università degli Studi “G. d’Annunzio” Chieti-Pescara, Via dei Vestini 31, 66100 Chieti, Italy; 3 Laboratorio de Investigación en Productos Naturales, Facultad de Química y Farmacia, Universidad de El Salvador, Final Av. de Mártires y Héroes del 30 de Julio, San Salvador 1101, El Salvador; ulises.guardado@ues.edu.sv (U.G.C.); morena.martinez@ues.edu.sv (M.L.M.); marvin.nunez@ues.edu.sv (M.J.N.); 4 Museo de Historia Natural de El Salvador, Ministerio de Cultura, San Salvador 1101, El Salvador; jmenjivar@cultura.gob.sv

**Keywords:** *Maytenus chiapensis*, Celastraceae, quinonemethide triterpenoids, pristimerin, tingenone, HPLC-PDA

## Abstract

**Background**: Quinonemethide triterpenoids, known as celastroloids, constitute a relatively small group of biologically active compounds restricted to the Celastraceae family and, therefore, they are chemotaxonomic markers for this family. Among this particular type of metabolite, pristimerin and tingenone are considered traditional medicines in Latin America. The aim of this study was the isolation of the most abundant celastroloids from the root bark of *Maytenus chiapensis*, and thereafter, to develop an analytical method to identify pristimerin and tingenone in the Celastraceae species. **Methods**: Pristimerin and tingenone were isolated from the *n*-hexane-Et_2_O extract of the root bark of *M. chiapensis* through chromatographic techniques, and were used as internal standards. Application of a validated RP HPLC-PDA method was developed for the simultaneous quantification of these two metabolites in three different extracts, *n*-hexane-Et_2_O, methanol, and water, to determine the best extractor solvent. **Results**: Concentration values showed great variation between the solvents used for extraction, with the *n*-hexane–Et_2_O extract being the richest in pristimerin and tingenone. **Conclusions**: *M. chiapensis* is a source of two biologically active quinonemethide triterpenoids. An analytical method was developed for the qualification and quantification of these two celastroloids in the root bark extracts of *M. chiapensis.* The validated method reported herein could be extended and be useful in analyzing Celastraceae species and real commercial samples.

## 1. Introduction

Species of the Celastraceae family have had a long history in traditional medicine and agriculture in North Africa, South and Central America, and Central and East Asia [1]. The therapeutic potential of Celastraceae species has been mainly attributed to the presence of quinonemethide triterpenoids (QMTs), a group of triterpenoids with unique structural features [2]. QMTs contain a *D:A*-friedo-nor-oleanane skeleton characterized by a particular oxygenation pattern with an unsaturated system involving rings A and B, and the majority of them bear a highly oxidized ring E [2]. QMTs constitute a relatively small group of biologically active compounds restricted to the Celastraceae family, commonly referred to as the bittersweet family [3] and, therefore, they are considered to be chemotaxonomic markers for this family. For this reason, QMTs and their structurally related congeners, phenolic triterpenoids, and triterpene dimers and trimers, were given the general name celastroloids by Brüning and Wagner [4]. QMTs have been reported mainly in the *Maytenus* [5], *Celastrus* [6], and *Tripterygium* [7] genera. This particular class of naturally occurring products, which are exclusively accumulated in the root barks of the plants that contain them, show a wide range of bioactivities, including cytotoxic [8,9], anti-inflammatory [10], antioxidant [11], antimicrobial [12], antiparasitic [13], and insecticidal [14] properties. 

Since 1936, when celastrol, the most extensively studied quinonemethide, was isolated from *Tripterygium wilfordii* [15], a variety of QMTs have been reported from Celastraceae species. In particular, pristimerin and tingenone, isolated for the first time from *Pristimera indica* [16] and *Euonymus tingens* [17], respectively, are the most frequently reported celastroloids. These two naturally occurring quinonemethide triterpenoid orange pigments are traditional medicines derived from the Celastraceae family and have long been used for the treatment of a variety of ailments [3,7]. Pristimerin has been reported to have promising clinical potential as both a therapeutic and chemopreventive agent for various types of cancer, including breast [18], glioma [19], prostate [20], pancreatic [21], ovarian [22], colon [23], esophageal squamous [24], osteosarcoma [25], and uveal [26] cancer, via a number of mechanisms [27]. Moreover, tingenone displays antinociceptive [28] and antiprotozoal [29] activities. Since there is pharmacological interest in this type of metabolite and their synthesis is not commercially viable, some research groups have investigated in vitro plant systems to increase their production [30].

In the course of the search for bioactive metabolites from species of the Celastraceae family, phytochemical studies on *Maytenus chiapensis*—a Celastraceae species collected in El Salvador and commonly named “Escobo blanco”—have reported the isolation of sesquiterpenoids [31,32,33] and tetracyclic and pentacyclic triterpenoids [34,35,36] from the areal parts of the plant. 

Taking into consideration the relevance of QMTs from a chemotaxonomic and therapeutic point of view, the aim of our study is to develop a validated analytical method to identify pristimerin and tingenone in the root barks of Celastraceae species. To perform this task and to investigate the previously unreported pristimerin and tingenone content in *M. chiapensis* root bark, the two known QMTs were isolated, characterized, and subsequently used as pure standard samples in HPLC analysis. Moreover, the successful application of a validated RP HPLC-PDA method is developed for the qualification and quantification of these two pharmacologically relevant QMTs in *M. chiapensis* extracts. Three different solvents were used to optimize the extraction procedure of the QMTs under study. The validated method reported herein could be extended and be useful in analyzing commercial samples.

## 2. Materials and Methods

### 2.1. Chemical

The solvents, methanol (HPLC-grade), water (HPLC-grade), *n*-hexane, diethyl ether, dichloromethane, and chloroform, and formic acid were purchased from Sigma-Aldrich (St. Louis, MO, USA) and used without further purification. Pristimerin and tingenone were isolated from the root bark of *Maytenus chiapensis* and used as pure standards (purity ≥99%) after their NMR characterization (see Figures S1 and S2 in the Supplementary Material).

### 2.2. Plant Material

The root bark of *Maytenus chiapensis* Lundell (Celastraceae) was collected at Montecristo National Park (latitude: 14°23′39′′ N, longitude: 89°23′10′′ W, elevation: 1617 msnm) in the municipality of Metapán, Santa Ana, El Salvador, in March 2018, and was identified by Jenny Elizabeth Menjívar Cruz, curator of the Herbarium at the Museo de Historia Natural de El Salvador. A voucher specimen (J. Menjívar et al. 4255) was deposited in the Herbarium at the Museo de Historia Natural de El Salvador, El Salvador.

### 2.3. Extraction and Isolation of Pristimerin and Tingenone

The root bark (650 g) of *M. chiapensis* was extracted with *n*-hexane–Et_2_O in a Soxhlet apparatus as previously reported [37]. The extract (27.2 g) was chromatographed on Sephadex LH-20 (*n*-hexane–CHCl_3_–MeOH, 2:1:1) to afford 15 final fractions after combination on the basis of their TLC profile. Fractions 7 and 8, after successive chromatographies on Sephadex LH-20 (*n*-hexane–CHCl_3_–MeOH, 2:1:1), silica gel (CH_2_Cl_2_–Et_2_O of increasing polarity), and preparative HPTLC developed with *n*-hexane–Et_2_O (4:6), gave rise to pristimerin (680 mg, R*_f_* 0.35) and tingenone (210 mg, R*_f_* 0.56). Their structures were identified by comparison of their ^1^H and ^13^C NMR (Bruker Avance 500 spectrometer, Bruker, Billerica, MA, USA) and MS (Micromass Autospec spectrometer, Micromass, Manchester, UK) data with those previously reported [38]. 

### 2.4. Preparation of Plant Extracts for HPLC Analysis

The methanolic and *n*-hexane–Et_2_O (1:1) extracts were prepared by dissolving 2.5 g of dried powdered root bark into 100 mL of organic solvents, and afterwards macerated for 72 hours at 25 °C. Both extracts were concentrated under reduced pressure at 40 °C to obtain 700 mg and 350 mg of crude residues, respectively.

The water extraction was carried out by dissolving 2.5 g of powdered root bark with 100 mL of water by magnetic stirrer ultrasonic (VWR, model 97043-988, operating frequency at 35 kHz) for 90 min at 25 °C. The aqueous extract was further filtered in Whatman No. 91 paper. The filtrate was frozen at −20 °C in an ultra-low temperature freezer (Fischer Scientific, Waltham, MA, USA) and lyophilized in a lyophilizator under 0.1 mmHg pressure at −50 °C (Labconco, Freezone, Kansas City, MO, USA) for 72 h. The resulting powder (130 mg) was stored at −20 °C until used.

### 2.5. HPLC-PDA Apparatus and Conditions

The chromatographic system consisted of an Alliance W2690 separation module equipped with an online degasser, an automatic injector, and a W2487 photodiode array detector set at 420 nm for the detection of pristimerin and tingenone. Data were collected and processed using Empower v.2 software for HPLC system (Waters, Milford, MA, USA). Separation was performed with a column Supelco Ascentis RP C18 (150 mm × 4.6 mm; particle size 5 µm, Sigma-Aldrich, St. Louis, MO, USA) column equipped with a Sentry Guard Cartridge (3.9 mm x 20 mm; Waters, Sigma-Aldrich, St. Louis, MO, USA) guard column. Both columns were maintained at 25 ± 1 °C. The mobile phase consisted of water with 0.4% of formic acid (v/v) (solvent A) and methanol (solvent B), using a gradient elution program for 10 min with a flow rate of 1.2 mL/min as follows: linear gradient ratio A/B 90:10 from the beginning of the chromatographic run to 6.0 min, A/B 90:10 to A/B 70:30 gradient from 6.01 min to 10.0 min, and finally, linear gradient ratio A/B 90:10 from 10.01 min to 15.0 min. 

### 2.6. Preparation of Samples

A total of 10.0 mg of each extract was dissolved in 10 mL of methanol, using an ultrasonic bath (VWR, model 97043-988, operating frequency at 35 kHz) at room temperature. The sample solutions were filtrated through a 0.22 µm membrane filter before being subjected to HPLC analysis. 

### 2.7. Method Validation

#### 2.7.1. Calibration, Linearity and Quality Control Samples

Standard solutions of pristimerin and tingenone (quality control samples, QC samples) were prepared in methanol at a concentration of 1000 µg/mL. Combined working solutions of mixed standards (QC samples) at concentrations of 10, 15, 30, 50, 80, and 100 µg/mL were obtained by the dilution of mixed stock solutions at 1000 µg/mL in a volumetric flask containing methanol. Calibration curves obtained at 420 nm were plotted using a weighted linear least-squares regression analysis. Concentrations of the QMTs (QC samples) were calculated by interpolating their peak areas on the calibration curve. The linearity of the investigated compounds was obtained by using the plant extract samples spiked at different concentrations. However, the slopes obtained from samples were different from those of the standard solution.

#### 2.7.2. Limit of Detection (LOD) and Limit of Quantification (LOQ)

The quality control samples (QC) were prepared to determine the limit of quantification (LOQ), the intra- and inter-assay precision and accuracy of the method, and defined according to International Guidelines, International Conference on Harmonisation (ICH) Q2 (R1). QC samples at three different concentration levels (QC low = 15.0, QC medium = 50.0, and QC high = 80.0 µg/mL) were used to validate the analytical method. The limit of detection (LOD) was calculated from the calibration graphic and was defined as 3 times the standard deviation of blank samples divided by the analytical sensitivity. The LOQ was defined as the lowest concentration on the calibration curve, which could be measured (*n* = 5) with a precision (RSD%) not exceeding 20% and with an accuracy between 80% and 120%. The method’s efficiency was measured by comparing the peak areas obtained from several samples obtained using pretreatment extraction processes and different extraction solvent systems. Analysis of these results allowed for an evaluation of the best extraction procedures, leading to the maximum recovery for the cited metabolites, minimizing solvent consumption and time.

## 3. Results and Discussion

### 3.1. Isolation of Pristimerin and Tingenone from Maytenus chiapensis

**Figure 1:**
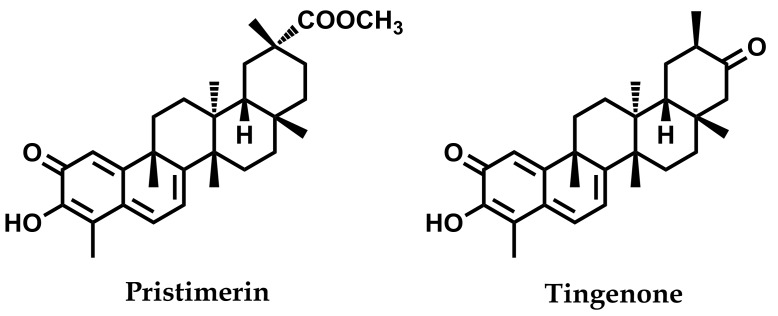
Structure of the main quinonemethide triterpenoids isolated from the root bark of *Maytenus chiapensis.*

Following the methodology previously established in our laboratory [37], multiple chromatographic steps of the root bark extract (*n*-hexane–Et_2_O, 1:1, 27.2 g) of the plant were carried out to yield pristimerin (680 mg) and tingenone (210 mg). The structures of these two known quinonemethide triterpenoids (Figure 1) were identified by comparison of their spectroscopic and spectrometric data with values reported in the literature [38] (see Figures S1 and S2 in the Supplementary Material). Following this, these compounds were used as internal standards in the HPLC analysis.

### 3.2. HPLC Analysis

The relevance of QMTs from a chemotaxonomic and therapeutic point of view has led several research groups to study their content in Celastraceae species, and some HPLC analyses have been reported. Thus, an HPLC method for the quantification of quinonemethide derivatives of five Brazilian morphological types of *Maytenus ilicifolia* has been reported [39]. Some years later, analysis by HPLC-DAD of *M. ilicifolia* extracts from root barks of adult plants and roots of seedlings indicated that pristimerin is the major component of both extracts [11]. Nossack and co-workers quantified pristimerin and tingenone (maitenin) in hydroalcoholic and aqueous extracts from the leaves and root bark of *Maytenus aquifolium* (“espinheira santa”) by HPLC-UV coupled with mass spectrometry (LC-MS) as a procedure for assessing the quality of this phytomedicine [40]. Moreover, a simple HPLC method was developed for the identification and comparison of quinonemethide triterpenes in wild *Hippocratea excelsa* and “cancerina”, a method useful for the control of this herb used in Mexican traditional medicine as an alternative cancer treatment [41]. In addition, Roca-Mézquita and co-workers performed quantitative analysis of pristimerin and tingenone in a dichloromethane extract of *Elaeodendron trichotomum*, revealing that this species contains both celastroloids, although, unexpectedly, pristimerin was present in very low concentration [29]. 

In the current study, the successful application of a validated RP HPLC-PDA method was performed for the qualification and quantification of pristimerin and tingenone in three different extracts of *Maytenus chiapensis*, using different solvents to optimize the extraction procedure. To carry out this task, a gradient mobile phase had been tested in order to obtain separation of both QMTs. The Supelco Ascentis C18 column was chosen owing to the good separation with respect to peak symmetry, resolution, and total analysis time. A mobile phase system consisting of water with 0.4% of formic acid (v/v) (solvent A) and methanol (solvent B) was used. Under these conditions, the peak retention times of pristimerin and tingenone were 6.04 (± 0.4) min and 2.25 (± 0.3) min, respectively (see Figure S3 in the Supplementary Material). 

Calibration curves, obtained at 420 nm, were plotted using weighted (1/x^2^) linear least-squares regression analysis. The calibration curves were linear over the concentration range tested, with the coefficient of determination *r*^2^ ≥ 0.9981 as reported in Table 1. The within-assay precision (repeatability) of the method was determined by performing three consecutive assays in the same day on QMTs samples spiked at three different standard concentration levels, i.e., 15 (low level), 50 (medium level), and 80 µg/mL (high level), which are within the range of the calibration curve. The results obtained are shown in Table 2.

### 3.3. Simultaneous Quantification of Pristimerin and Tingenone

HPLC analysis was used for the simultaneous determination of pristimerin and tingenone from *M. chiapensis* root bark in different solvents. Triplicate measurements were performed to determine the mean amount of both metabolites. The results indicate the influence of the solvent used in the extraction process (Table 3). A mixture of *n*-hexane–Et_2_O (1:1) was the best extractor solvent since it extracted the highest quantity of pristimerin and tingenone, whereas water was the worst extractor solvent as neither of the two compounds could be detected.

This study revealed that *Maytenus chiapensis* is a source of pristimerin and tingenone, two components with great potential in drug development. Moreover, an RP HPLC-PDA method was developed and validated for their quantification in root bark extracts of this species. The results indicate that a mixture of *n*-hexane–Et_2_O (1:1) is the optimal extractor solvent for these two promising bioactive naturally occurring compounds. The methodology described herein could be extended to other Celastraceae species, and be useful in the analysis of commercial samples. 

## Figures and Tables

**Table 1:** Mean linear calibration curve parameters obtained by weighted linear least-squares regression analysis of three independent six non-zero concentration points.

Compound	Linearity Range (µg/mL)	Slope	Intercept	Determination Coefficient (*r*^2^)
**Pristimerin**	1–100	74653–79342	−19550 to −4325	0.9981
**Tingenone**	1–100	45234–49342	−2345 to 13456	0.9990

**Table 2:** Assay precision (RSD%) and trueness (bias%) of the analytical method obtained from the analysis of quinonemethide triterpenoids (QMTs) samples.

Parameters	Pristimerin	Tingenone
**Theoretical *^a^***	**15.0**	
**Mean back-calculated *^a^***	14.90	15.02
**RSD%**	4.45	2.34
**Bias%**	1.45	2.65
**Theoretical *^a^***	**50.0**	
**Mean back-calculated *^a^***	50.10	49.80
**RSD%**	4.60	2.80
**Bias%**	1.80	2.10
**Theoretical *^a^***	**80.0**	
**Mean back-calculated *^a^***	89.87	88.99
**RSD%**	5.76	6.42
**Bias%**	4.56	4.1

*^a^* Concentration expressed as µg/mL

**Table 3:** Pristimerin and tingenone content in *M. chiapensis* root bark.

Solvent	Extraction Method	Content in Pristimerin			Content in Tingenone		
		µg/mL *^a^*	mg/g Extract	mg/g dry Material	µg/mL	mg/g Extract	mg/g Dry Material
*n*-hexane–Et_2_O (1:1)	Maceration	46.41 ± 0.10	46.41 mg	6.50 mg	31.66 ± 0.15	31.66 mg	4.43 mg
Methanol	Maceration	18.01 ± 0.20	18.01 mg	5.04 mg	10.15 ± 0.18	10.15 mg	2.84 mg
H_2_O	UAE *^b^*	ND *^c^*	ND *^c^*	ND *^c^*	ND *^c^*	ND *^c^*	ND *^c^*

*^a^* Data are expressed as µg/mL of dry plant; *^b^* UAE: ultrasound-assisted extraction; *^c^* ND: not detected.

## Supplementary Materials

The following are available online at https://www.mdpi.com/2305-6320/6/1/36/s1, Figure S1: ^1^H and ^13^C NMR spectra of pristimerin in CDCl_3_ (500 and 125 MHz, respectively), Figure S2: ^1^H and ^13^C NMR spectra of tingenone in CDCl_3_ (500 and 125 MHz, respectively), Figure S3: HPLC chromatograms with UV detection at 420 nm of (A) standard compounds, tingenone and pristimerin, and (B) *n*-hexane–Et_2_O (1:1) extract (for chromatographic protocol, see Experimental section).
