# Supplementary material for: Development and Validation of an HPLC-PDA Method for Biologically Active Quinonemethide Triterpenoids Isolated from Maytenus chiapensis
Source: Medicines (Basel). 2019 Mar 7;6(1):36. doi: 10.3390/medicines6010036 (PMC6473362; doi:10.3390/medicines6010036)
Supplement: Supplementary file 1 [file medicines-06-00036-s001.pdf]

# Supplementary Materials: Development and Validation of an HPLC-PDA Method for Biologically Active Quinonemethide Triterpenoids Isolated from *Maytenus chiapensis*

**Vito Alessandro Taddeo, Ulises Guardado Castillo, Morena Lizette Martínez, Jenny Menjivar, Ignacio Antonio Jiménez, Marvin José Núñez and Isabel López Bazzocchi**

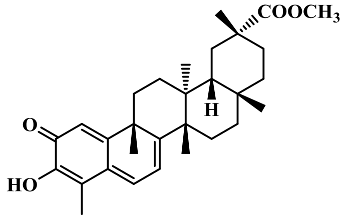


**Pristimerina**

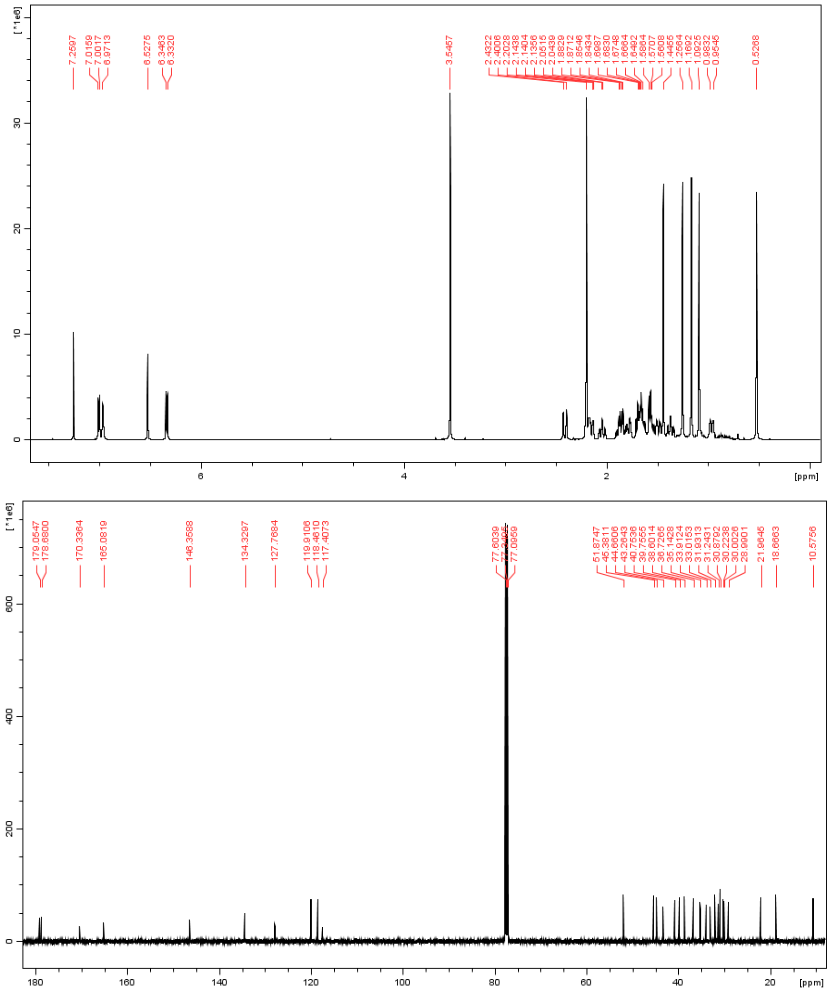

**Figure S1.** $^1H$ and $^{13}C$ NMR spectra of pristimerin in $CDCl_3$ (500 and 125 MHz, respectively).

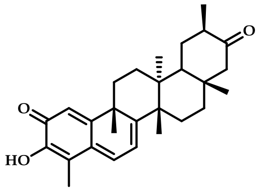

**Tingenona**

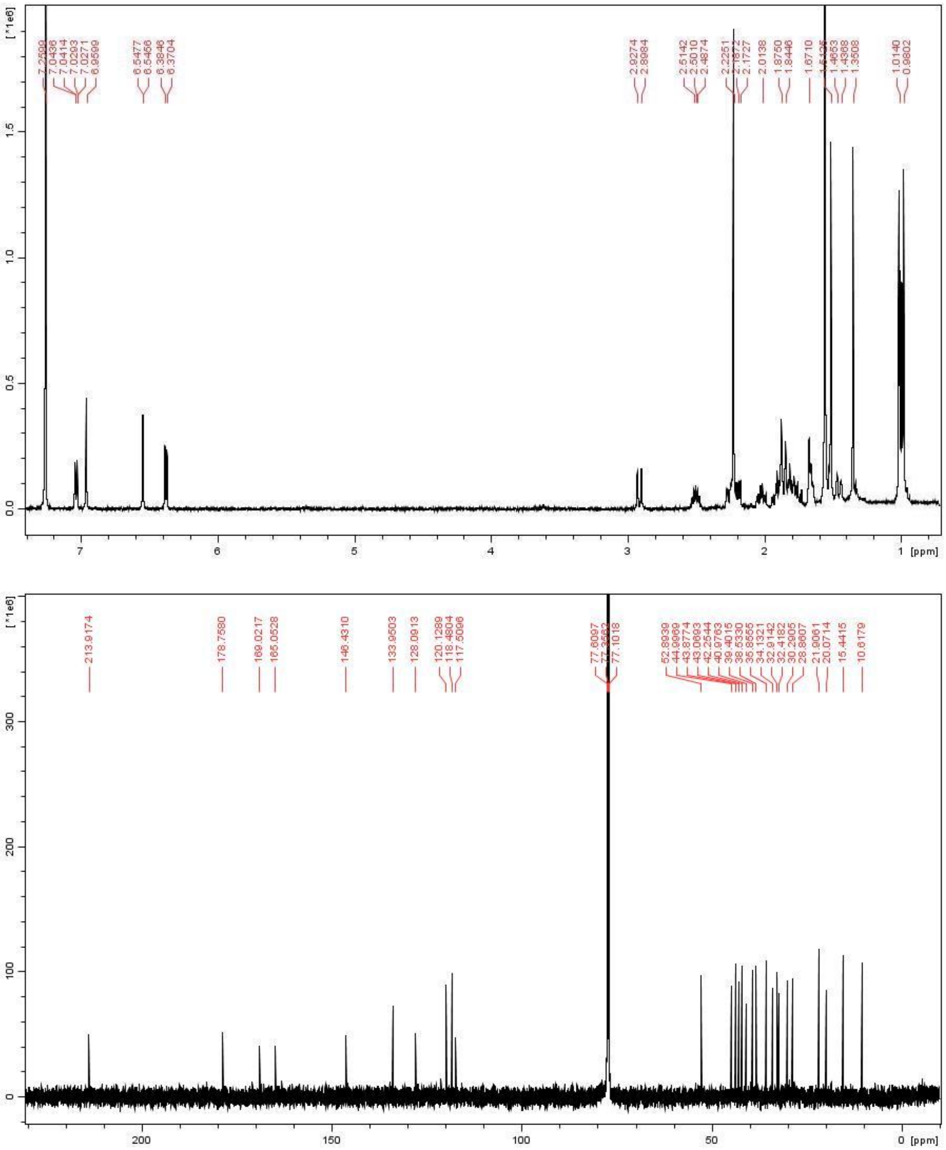

**Figure S2.** $^{1}H$ and $^{13}C$ NMR spectra of tingenone in $CDCl_3$ (500 and 125 MHz, respectively).

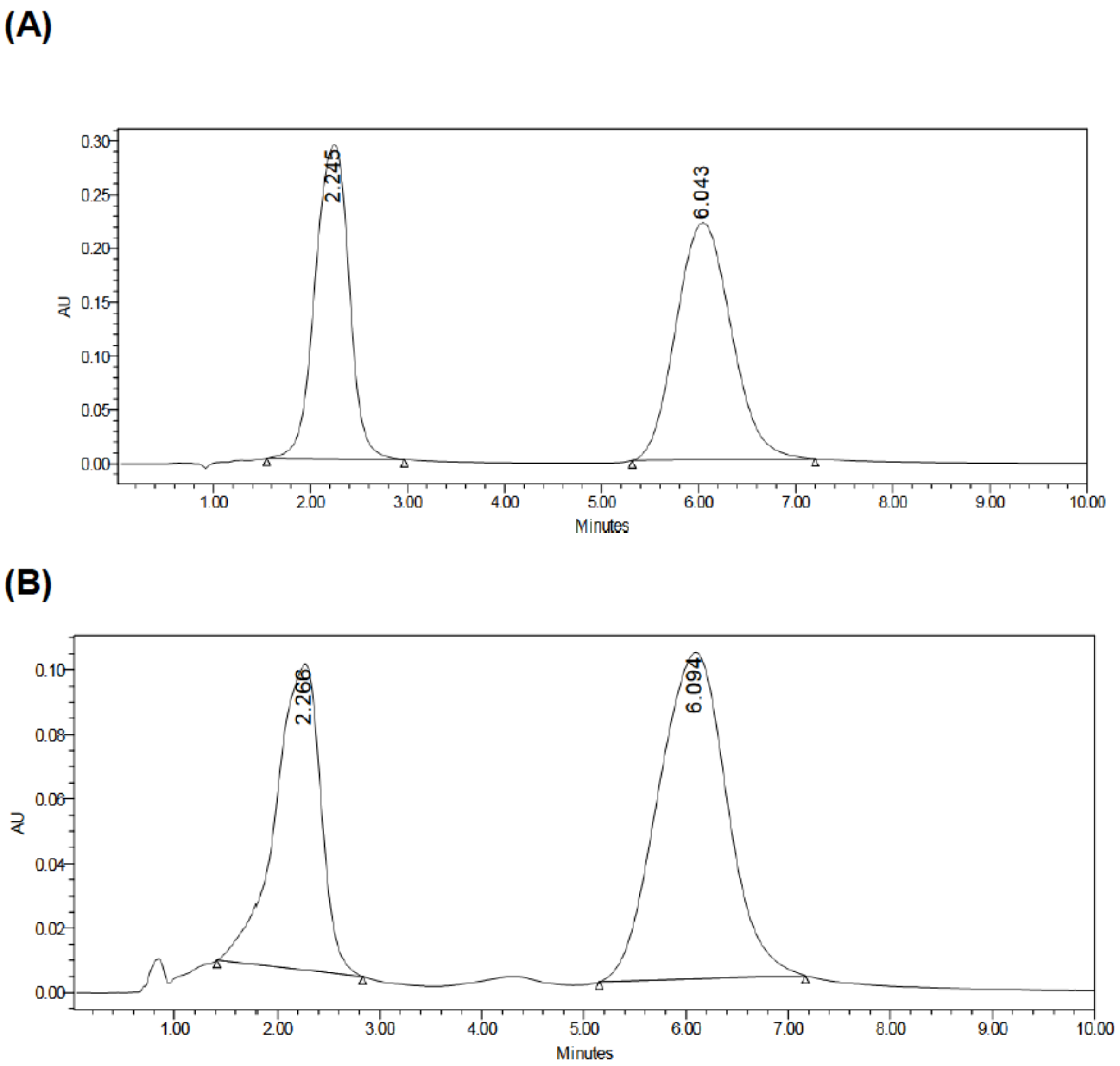


**Figure S3.** HPLC chromatograms with UV detection at 420 nm of (**A**) standard compounds, tingenone and pristimerin, and (**B**) *n*-hexane–$Et_2O$ (1:1) extract (for chromatographic protocol, see Experimental section).
